# Role of psychotherapy on antenatal depression, anxiety, and maternal quality of life: A meta-analysis

**DOI:** 10.1097/MD.0000000000020947

**Published:** 2020-07-02

**Authors:** Caixia Li, Xiaohua Sun, Qing Li, Qian Sun, Beibei Wu, Dongyun Duan

**Affiliations:** Department of Obstetrics, Maternity and Child Health Care of Zaozhuang, Zaozhuang, Shandong, PR China.

**Keywords:** cognition, fear of birth, gynecology, obstetrics, perinatal, psychotherapy

## Abstract

**Background:**

Depression and anxiety are common psychological manifestations encountered during the antenatal stage of pregnancy. Treatments by pharmacological interventions have been reported to impart negative implications on maternal and fetal health outcomes. Therefore, the use of psychotherapeutic interventions to bypass these side-effects and manage depression, anxiety has received a lot of attention. A meta-statistical consensus regarding the intervention is available, but with several limitations. In this study, we attempt to address these limitations and provide the current state of evidence evaluating the influence of psychotherapy on antenatal depression, anxiety, and maternal quality of life.

**Objective:**

To demonstrate the effects of psychotherapy on depression, anxiety, and maternal quality of life during the antenatal stage of pregnancy.

**Methods:**

A systematic identification of literature was performed according to PRISMA guidelines on four academic databases: MEDLINE, Scopus, EMBASE, and CENTRAL. A meta-analysis evaluated the influence of psychotherapy on depression, anxiety, and maternal quality of life as compared to conventional obstetric care.

**Results:**

Out of 1146 records, 22 articles including 2146 pregnant women (mean age: 28.6 ± 2.8 years) were included in this review. This systematic review presents a 1b level of evidence supporting the use of psychotherapy for reducing depression, anxiety and enhancing maternal quality of life. The meta-analysis reveals the beneficial effects of psychotherapy for reducing depression (Hedge g: −0.48), anxiety (−0.47) and enhancing maternal quality of life (0.19) as compared to conventional obstetric care.

**Conclusions:**

The current systematic review and meta-analysis recommend the use of psychotherapy as for reducing depression, anxiety and enhancing maternal quality of life during the antenatal stage of pregnancy.

## 1 Introduction

The prominence of depression and anxiety during the antenatal stage of pregnancy is extremely high.^[1]^ According to the recent World Health Organization statistics, depression and anxiety are a public health concern for women of childbearing age.^[2,3]^ Recent epidemiological studies suggest that approximately 10% to 15% of all pregnant women suffer from depression and anxiety related disorders.^[4,5]^ This increased prevalence of anxiety and depression can also hint towards the rising suicidal tendencies amongst mothers and their partners undergoing the bereavement.^[6]^ Moreover, this incidence has been reported to be even higher in developing countries (for a detailed review see^[2]^).

The literature suggests multiple mechanisms due to which the onset of these psychological manifestations are high.^[7,8]^ For instance, a direct correlation has been reported between the pregnancy-related changes in the level of maternal hormones such as progesterone, estrogen, prolactin, estradiol with a shift in hypothalamus-pituitary axis^[9]^ and the levels of cortisol.^[7,10,11]^ Pompili et al,^[11]^ for instance, suggested that the levels of prolactin and thyroid are usually dysregulated during the antenatal stage of pregnancy and that it can be associated with suicidal attempts due to its complex compensatory role in correcting the central serotonin activity. The authors also mentioned that the evaluation of the hormonal levels of thyroid, prolactin is of great importance owing to their ability predict suicidal attempts. Similarly, changes in epigenetic mechanisms due to varying antenatal conditions have been shown to act as a supplementary co-factor promoting depression, and anxiety.^[12,13]^ A recent review by Wesołowska et al^[14]^ mentioned that in addition to the aforementioned factors a range of environmental, that is, martial, family,^[15,16]^ and socioeconomic factors,^[17,18]^ could also act as additional precursors for the development of these disorders.

Together, these manifestations have been reported to impart a wide range of negative implications on both maternal and fetal health outcomes.^[19,20]^ In terms of maternal health, high levels of antenatal anxiety (fear of childbirth), depression have been associated with higher incidences of cesarean section,^[21]^ premature delivery,^[22]^ pregnancy-related complications, that is, anemia, preeclampsia,^[19,20]^ and poorer maternal quality of life.^[23]^ Furthermore, a strong correlation has been reported between the prominence of antenatal depression with higher incidences of postnatal depression, and poor maternal-fetal attachment.^[24–26]^ Despite the medical advancements in the past decades and the development of numerous novel interventions,^[27]^ epidemiological studies show no sign of decline in the onset of depression and anxiety during the antenatal stage of pregnancy.^[28]^ The main reason behind this could be negligence. Atif et al^[29]^ in their review mentioned that primary health care programs tend to focus more on maternal physical health as compared to mental health.

Conventionally, pharmacological interventions are considered as the front-line management approaches to alleviate depression and anxiety during pregnancy.^[30,31]^ Uguz et al,^[31]^ for instance, recommended the use of pharmacological prophylaxis while using serotonin-reuptake inhibitors to prevent the onset of depression and anxiety during the antenatal stage of pregnancy. The authors however, failed to discuss the negative implications of these drugs on fetal and neonatal health outcomes.^[32]^ Newport et al^[33]^ mentioned that irrespective of the class of the administered antidepressants, all drugs cross the placental barrier,^[34]^ and are present in both the amniotic fluid,^[35]^ and breast milk.^[36]^ Thereby, causing widespread changes in the fetal and neonatal health-related outcomes.

Taking this into consideration the use of psychotherapy to bypass these negative complications and simultaneously manage these psychosomatic disorders has received a lot of attention.^[33,37,38]^ Several underlying mechanisms have been reported to support the favorability of this approach. For instance, studies have demonstrated that psychotherapeutic interventions can enhance cognitive flexibility,^[39]^ maternal motivation,^[40]^ allow self-regulation of thoughts,^[41]^ and restructuring of negative emotions.^[42]^ A meta-analysis by Farrand and Woodford^[43]^ confirmed that interventions by psychotherapeutic techniques can not only benefit the mental health status but also provide enhancements in physical health status and quality of life. Ekers et al,^[44]^ in addition, reported that the impact of psychotherapeutic intervention can be augmented by promoting its delivery through paraprofessionals such as nurses. The authors mentioned that the cost-effectiveness,^[45]^ cultural proximity,^[46]^ and nurse-mother bonding,^[47]^ might further the effects of the psychotherapeutic interventions to alleviate depression and anxiety.

To date, two systematic reviews have evaluated the effects of psychotherapy on antenatal depression and anxiety.^[48,49]^ However, a few limitations persisted in these review studies that raise questions concerning the reliability of their results. Firstly, the findings from Smith et al^[48]^ are misleading. The authors analyzed the effects of mindfulness-based therapies on antenatal depression and anxiety. These approaches do not quite represent the conventional psychotherapeutic interventions commonly employed at the psychiatric care units. Secondly, van Ravesteyn et al^[49]^ analyzed the effects of psychotherapy on depression and anxiety. Again, this review misleads the analyses for anxiety as only one study was incorporated by the reviewers in the analyses. Moreover, the article included very few numbers of trials i.e. seven studies for cognitive psychotherapy. This could be because of the strict inclusion criteria imposed by the reviewers. Another reason why this review needs to be updated is that since its publication in 2017, several high quality randomized controlled trials have been published.^[50–53]^ Therefore, warranting the need for an updated systematic review and meta-analyses.

This systematic review and meta-analyses will attempt to address this gap in the literature by assessing the role of psychotherapeutic interventions on antenatal depression, anxiety, and maternal quality of life.

## 2 Methods

This systematic review and meta-analysis were carried in adherence to PRISMA guidelines.^[54]^ A PRISMA checklist has been provided in the Supplementary file. The ethical approval was not necessary because it is a systematic review and meta-analysis.

### 2.1 Data search strategy

We searched four academic databases (MEDLINE, CENTRAL, EMBASE, and Scopus) from inception until December 2019 using MeSH keywords “antenatal”, “pregnancy”, “perinatal”, “before-birth”, “pre-birth”, “psychotherapy”, “psychoanalytic therapy”, “counseling”, “cognitive therapy”, “behavioral therapy”, “cognitive behavioral therapy”, “CBT”, “psychoeducation”, “interpersonal therapy”, “crisis oriented therapy”, “anxiety”, “depression”, “fear of birth”, “fear of child birth”. In addition, we screened the bibliography of the included studies for any additional relevant study. The inclusion criteria for the studies were as follows:

1. Studies evaluated the efficacy of psychotherapy on depression, anxiety and quality of life outcomes during the antenatal stage of pregnancy.
2. Studies evaluated pregnant women in the antenatal stage of pregnancy.
3. Studies evaluated the depression, anxiety and/or maternal quality of life outcome through a valid and reliable assessment method (e.g. State trait anxiety inventory, Edinburg perinatal depression scale, fear of birth scale, pregnancy worry and stress questionnaire, Wijma delivery expectancy scale, phobia anxiety scales, WHO quality of life scale-BREF, European quality of life scale, quality of life, etc).
4. Studies were either randomized controlled trials, quasi randomized controlled trials, controlled clinical trials, prospective observational trials with control groups or retrospective trials.
5. Studies published in peer-reviewed scientific journals, conferences.
6. Studies published in the English language.

The exclusion criteria for the study included the following:

1. Studies that did not evaluate the efficacy of psychotherapy on depression, anxiety and quality of life during the antenatal stage of pregnancy were excluded.
2. Studies that did not evaluate pregnant women in the antenatal stage of pregnancy were excluded.
3. Studies that did not evaluate the aspects of depression, anxiety and/or maternal quality of life outcome through a valid and reliable assessment method were excluded.
4. Studies which were not case controlled studies were excluded.
5. Studies which were not published in peer-reviewed journals were excluded.
6. Studies published languages other than English were excluded.

The selection procedure was independently replicated by two reviewers to avoid biasing. The following data were extracted from the included studies: authors, sample description (gender, age), method of assessment, intervention, comparator, stage of assessment and outcome measures. In the articles where quantitative data outcomes were incomplete or not mentioned the reviewers made attempts to contact respective corresponding authors for additional data.

### 2.2 Quality assessment

The risk of bias in the included studies was assessed by Cochrane's risk of bias assessment tool for randomized controlled trials and non-randomized controlled trials, that is, ROBINS-I.^[55,56]^ The included studies were independently appraised by 2 reviewers. The appraisal was done based on the presence of low, high or unclear risk of bias. The thresholds for interpretation of Cochrane risk of bias tool's assessment according to Agency for Healthcare Research and Quality standards is either good quality (all criteria are attained), fair quality (1 high-risk criteria or 2 unclear criteria) or poor quality (two or more criteria attained with high risks). Inadequate randomization, concealment of allocation and reporting of selective outcomes were considered as major threats for biasing.^[57]^ In cases of ambiguity, discussions were held between the reviewers until a consensus was reached. Moreover, a level of evidence analysis based on the Center for Evidence Based Medicine outcome was reported based on the type of included studies.^[58]^

### 2.3 Data analysis

A within group meta-analysis of the included studies was carried out using CMA (Comprehensive Meta-analysis version 2.0).^[59]^ The data was distributed and separately analyzed for depression, anxiety, and maternal quality of life. A meta-analysis was conducted based on a random effects model.^[60]^ The effect sizes are reported as weighted Hedge's g. The threshold for interpreting the weighted effect sizes are: ≤0.2 a small effect, ≤ 0.5 as a medium effect and ≥ 0.8 a large effect.^[61]^ Heterogeneity was assessed by computing I^2^ statistics. The threshold for interpreting heterogeneity is: 0% to 25% with negligible heterogeneity, 25% to 75% with moderate heterogeneity and ≥75% with substantial heterogeneity.^[62]^ Sensitivity analyses were performed in cases where substantial sources of heterogeneity persisted.^[63]^ Here, based on the presence or absence of inadequate randomization methods in the studies we either included or excluded the results of the studies. For each evaluated parameter details of weighted effect size, 95% confidence intervals, level of significance and heterogeneity have been duly reported. In addition, we analyzed publication bias by performing Duval and Tweedie's trim and fill procedure.^[64]^ This non-parametric method estimates the number of missing studies and computes the effect that these studies might have on the outcome of meta-analyses. Here, asymmetric studies are imputed from the left side of the plotted graph to identify the unbiased effect. Thereafter, these trimmed effects are refilled in the plotted graph and then the combined effect is recalculated. In the present review, the alpha level was set at 5%.

## 3 Results

**Figure 1:**
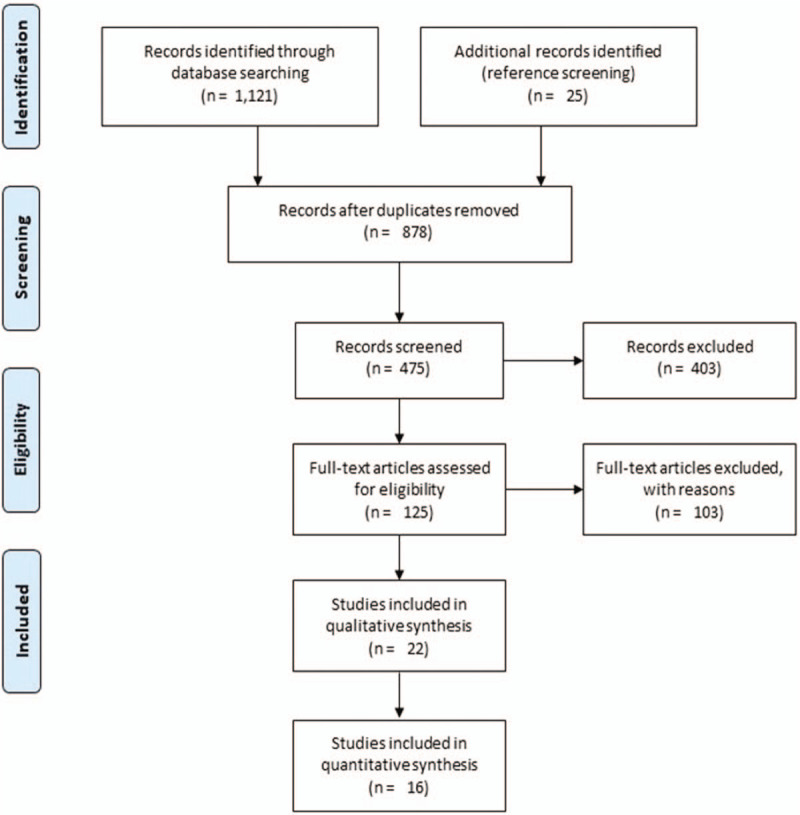
Illustrates the PRISMA flow chart for the included studies.

A preliminary search on 4 academic databases resulted in a total of 1121 studies, 25 more studies were included after the bibliography of these articles were screened (Fig. 1). Thereafter, upon excluding the duplicates and applying the inclusion criteria, a total of 22 studies were retained. In the included studies, 17 were randomized controlled trials,^[50–53,65–77]^ whereas five were controlled clinical trials.^[78–82]^ Significant reduction (*P* < .05) in depression and anxiety was reported in 18 of the included studies which received psychotherapy.^[52,53,65–78,80–82]^ and three studies reported no effect,^[50,51,79]^ of psychotherapy on depression, anxiety and maternal quality of life during the antenatal stage of pregnancy. Qualitative and quantitative data were then extracted from all the studies and summarized in Table 3 .

### 3.1 Risk of bias

#### 3.1.1 Randomized controlled trials

**Figure 2:**
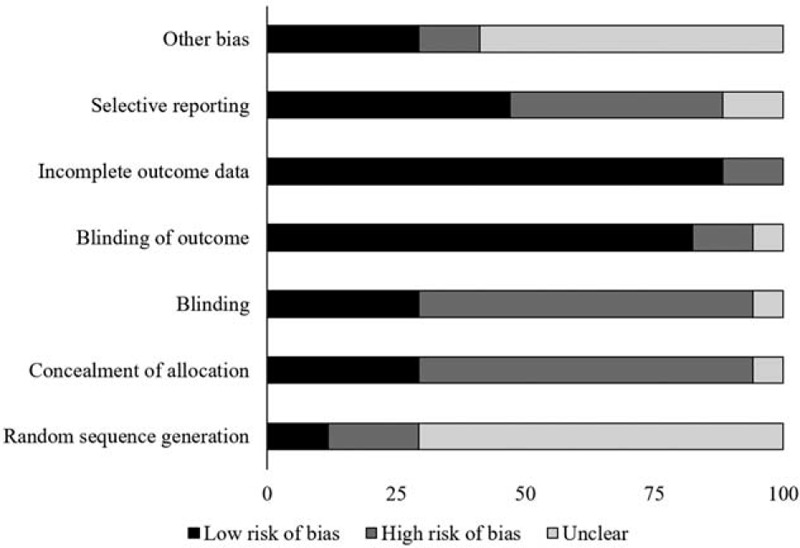
Illustrates risk of bias (%) within studies according to Cochrane risk of bias assessment tool for randomized controlled trials.

The risk of bias for the randomized controlled trials according to Cochrane's risk of bias assessment tool for randomized controlled trials has been demonstrated in Table 1. The overall risk in the included studies is poor. The highest risk of bias was observed to be due to lack of blinding of the participants, researchers, outcomes, sequence generation and other biases Figure 2. A level of evidence of 1b was observed for all the included studies based on their experimental design.

**Table 1:**
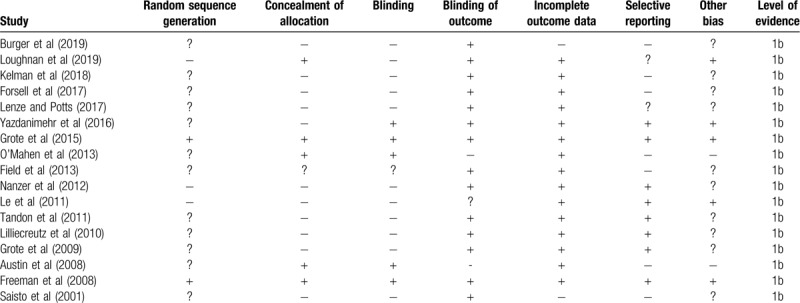
Illustrates the quality of the analyzed studies according to the Cochrane risk of bias assessment tool for randomized controlled trials (−: high risk of bias, +: low risk of bias, ?: unclear risk of bias.

#### 3.1.2 Controlled clinical trials

**Figure 3:**
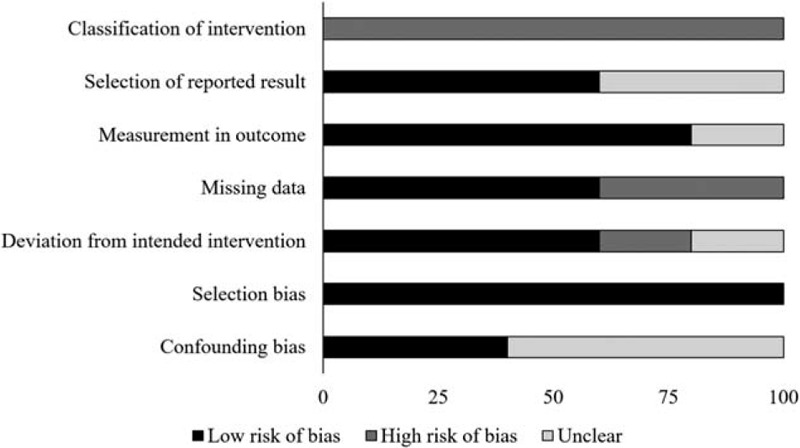
Illustrates risk of bias (%) within studies according to Cochrane risk of bias assessment tool for controlled clinical trials.

The prevalence of risk of bias for the controlled clinical trials according to Cochrane's risk of bias assessment tool for non-randomized controlled trials ROBINS-I has been demonstrated in Table 2. Here as well, the overall risk in the included studies is poor. The highest risk of bias was observed to be due to the lack of clarity in the confounding factors, classification of intervention and outcome measurement Figure 3. Furthermore, a few studies refrained from explaining the measures they undertook to manage missing data and/or analyses for intention to treat analysis. A level of evidence of 2b was observed for all the included studies based on their experimental design.

**Table 2:**
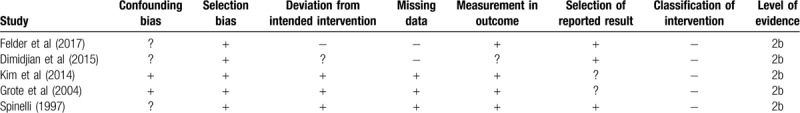
Illustrates the quality of the analyzed studies according to the Cochrane risk of bias assessment tool for non-randomized controlled trials ROBINS-I (-: high risk of bias, +: low risk of bias, ?: unclear risk of bias).

**Table 3:**
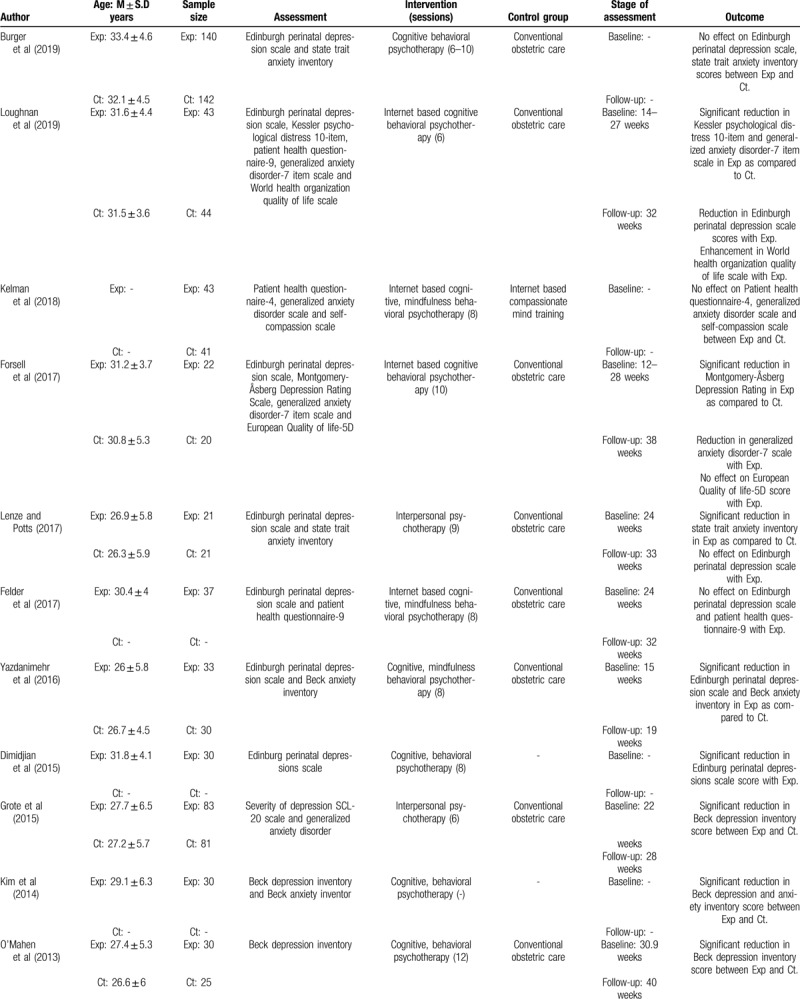
Illustrates the characteristics of the included studies.

**Table 3 (Continued):**
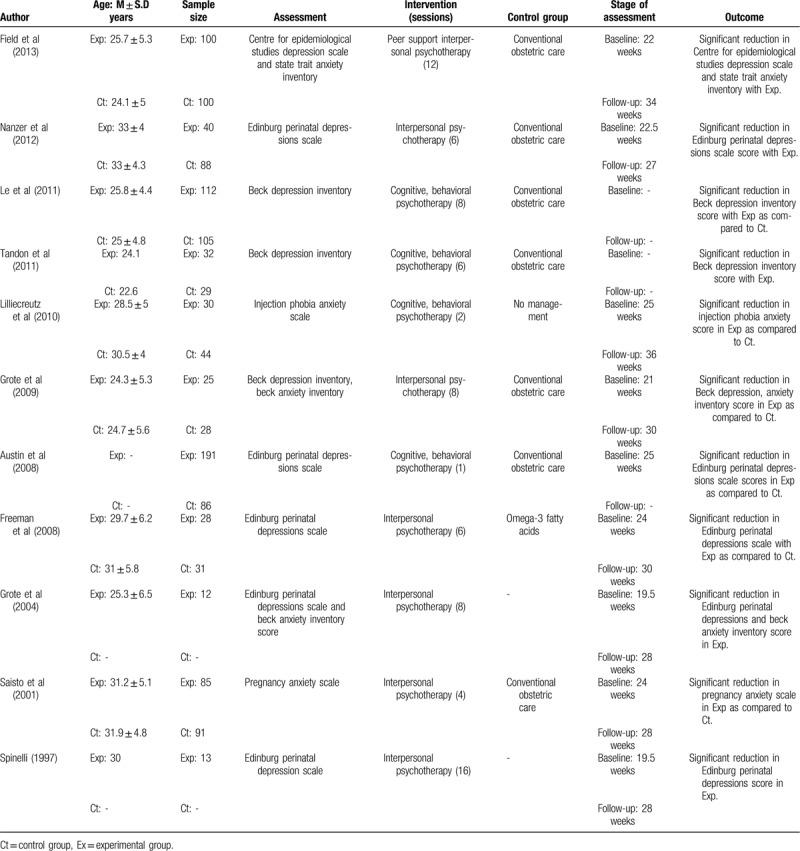
Illustrates the characteristics of the included studies.

#### 3.1.3 Publication bias

**Figure 4:**
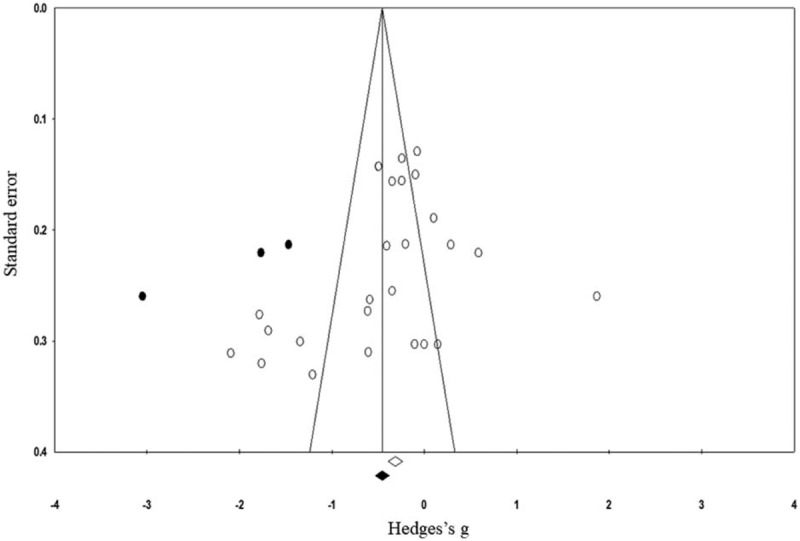
Illustrates the publication bias funnel plot by the Duval and Tweedie trim and fill procedure. Each of the analyzed effect is denoted by a circle in the plot. The boundaries of the plot mark the area where 95% of all the effects reside in case there were no publication biases. The vertical midline denotes the mean standardized effect of zero.

The trim and fill procedure identified three missing studies on the left side of the mean effect (Fig. 4). Further, according to random effect model, the point estimates and 95% confidence intervals for the evaluated parameters are −0.42 (−0.67 to −0.17). The trim and fill procedure report the imputed point estimate as −0.6 (−0.9 to −0.31).

#### 3.1.4 Participant information

A total of 2146 pregnant women were evaluated in the studies included in this review. Here, a total of 1180 women were a part of the experimental group where psychotherapy was administered, whereas 966 women were a part of the control group receiving conventional obstetric care. Two of the included studies did not mention the age of the included sample.^[51,65]^ However, from the studies that did report the age of their participants, the mean age of the included participants was 28.6 ± 2.8 years for the experimental and 28.2 ± 3.4 years for the control group.

#### 3.1.5 Assessment

Nine studies assessed the influence of psychotherapy on depression alone,^[65,68,71,73,74,76,78,79,82]^ whereas 2 studies assessed its influence on anxiety.^[72,75]^ Eleven studies jointly evaluated the effects of psychotherapy on both depression and anxiety.^[50–53,66,67,69,70,77,80,81]^ The average baseline, follow-up assessments for the included studies was performed at 23.2 ± 3.6 weeks, and 30.8 ± 5 weeks, respectively. However, from the included studies 6 did not report the stage at which they performed initial and follow-up assessments,^[50,51,71,76,78,81]^ whereas 1 study did not report the stage at which the follow-up assessment was performed.^[65]^

#### 3.1.6 Intervention

In the included studies, 13 used cognitive behavioral interventions,^[50,51,53,65,67,71,72,74,76–79,81]^ whereas nine of the studies used interpersonal psychotherapeutic measures.^[52,66,68–70,73,75,80,82]^ Moreover, all the included studies compared the effects of psychotherapy with conventional obstetric care except three studies.^[51,68,72]^

### 3.2 Meta-analysis reports

#### 3.2.1 Depression

**Figure 5:**
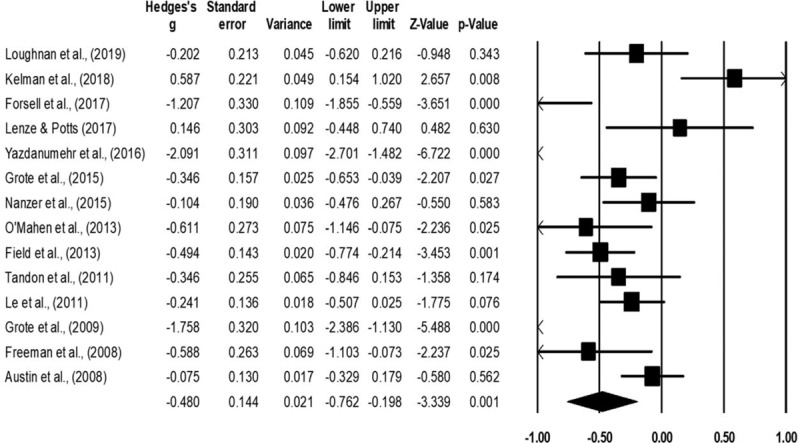
Illustrates the forest plot for studies evaluating the effects of psychotherapy on the outcome of depression during the antenatal stage of pregnancy. Weighted effect size is presented as boxes, 95% C.I are presented as whiskers. A negative effect represents a reduced outcome of depression; a positive effect represents an enhanced outcome of depression.

Depression was assessed in fourteen studies.^[51–53,65–71,73,74,76,77]^ Here, data from 803 participants was assessed in the experimental group receiving psychotherapy as compared to 729 in the control group. The assessment of depression was performed by 7 studies using Edinburg perinatal depression scale,^[52,53,65,67,68,73,77]^ three studies using Beck depression inventory,^[70,71,76]^ and one study each using self-efficacy questionnaire,^[51]^ severity of depression scale,^[69]^ and center for epidemiological studies depression inventory scale.^[66]^ An across group, random-effect analysis (Fig. 5) revealed a *medium* negative and significant effect of psychotherapy on depression as compared to conventional obstetric care (g: −0.48, 95% C.I: −0.76 to −0.19, *P* = .001) with moderate heterogeneity (I^2^: 39%).

#### 3.2.2 Anxiety

**Figure 6:**
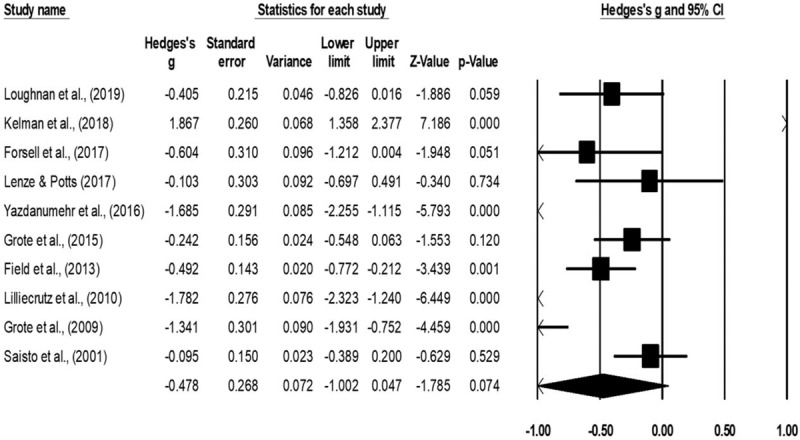
Illustrates the forest plot for studies evaluating the effects of psychotherapy on the outcome of anxiety during the antenatal stage of pregnancy. Weighted effect size is presented as boxes, 95% C.I are presented as whiskers. A negative effect represents a reduced outcome of anxiety; a positive effect represents an enhanced outcome of anxiety.

Anxiety was assessed in 10 studies.^[51–53,66,67,69,70,72,75,77]^ Here, data from 485 participants were assessed in the experimental group receiving psychotherapy as compared to 500 in the control group. The assessment of anxiety was performed by 4 studies each using generalized anxiety disorder,^[51,53,67,69]^ 2 studies using Beck anxiety inventory,^[70,77]^ and 1 study each using state trait anxiety inventory scale,^[52]^ pregnancy anxiety scale,^[75]^ injection phobia anxiety scale,^[72]^ and center for epidemiological studies anxiety inventory.^[66]^ An across group, random-effect analysis (Fig. 6) revealed a *medium* negative and significant effects of psychotherapy on anxiety as compared to conventional obstetric care (g: −0.47, 95% C.I: −1 to −0.04, *P* = .07) with moderate heterogeneity (I^2^: 32%).

#### 3.2.3 Maternal quality of life outcome

**Figure 7:**
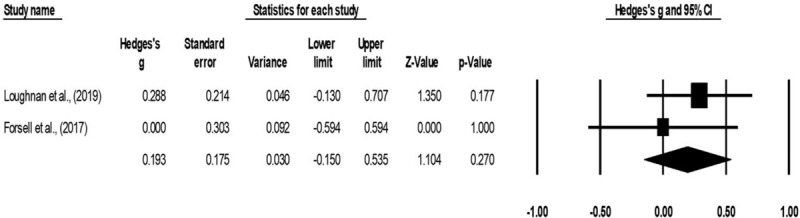
Illustrates the forest plot for studies evaluating the effects of psychotherapy on maternal quality of life outcome during antenatal stage of pregnancy. Weighted effect size is presented as boxes, 95% C.I are presented as whiskers. A negative effect represents a reduced EQ-5D score; a positive effect represents enhanced EQ-5D score.

Maternal quality of life outcome was assessed in 2 studies.^[53,67]^ Here, one study each used EQ-5D,^[67]^ and World health organization quality of life scale,^[53]^ to assess maternal quality of life outcome. Here, data from 65 participants was assessed in the experimental group receiving psychotherapy as compared to 64 in the control group. An across group, random-effect analysis (Fig. 7) revealed a *small* positive and non-significant effects psychotherapy on quality of life outcome as compared to conventional obstetric care (g: 0.19, 95% C.I: −0.1 to 0.5, *P* = .2) with no heterogeneity (I^2^: 0%).

## 4 Discussion

This review provides a 1b level of evidence regarding the beneficial effects of psychotherapy as compared to conventional obstetric care for reducing depression, anxiety and enhancing the maternal quality of life during the antenatal stage of pregnancy. Moreover, the present meta-analysis reveals the beneficial effects of psychotherapy for reducing depression (Hedge g: −0.48), anxiety (−0.47) and enhancing maternal quality of life (0.19) as compared to conventional obstetric care.

The use of psychotherapeutic interventions such as cognitive behavioral therapy and interpersonal therapy has been intensified in the past decade. David et al^[83]^ for instance, even termed these psychotherapeutic interventions as the gold standard approach to manage depression and anxiety during pregnancy.^[84,85]^ Most importantly, the use of this approach is favored because of its capability to avoid pharmacological complications.^[86]^ Cooper et al^[87]^ for instance, reported that the use of antidepressants such as selective serotonin-reuptake inhibitors during the antenatal stage of pregnancy could predispose towards poorer maternal health, quality of life and promote developmental fetal defects.^[88]^ Furthermore, the use of psychotherapeutic interventions has been preferred because of their ability to promote a challenging environment within which the pregnant women gain cognitive flexibility,^[39]^ and intrinsic-extrinsic motivation.^[89]^ Besides, the preference of psychotherapy has also been driven due to the intervention's ability to provide retainable effects. Dimidjian et al^[78]^ reported that training with a mindful cognitive behavioral task allowed a significant reduction in the depression scores which were maintained even during the 6-month postpartum phase. Likewise, studies also report that psychotherapy can promote the robustness of self-monitoring and attentional allocation cognitive resources, which might further allow a depressed and anxious woman to actively adapt according to a situation.^[90,91]^ Nagata et al^[39]^ mentioned that cognitive behavioral therapy can effectively combat situations where the loss of emotional automation is prevalent. Here, psychotherapy mediated enhancements in cognitive resources can allow a patient to replace their negative thoughts into objective prospects, thereby, providing a breakthrough from the vicious depression-anxiety cycle.^[92]^ In agreement with the existing state of literature, the current meta-analyses also report *medium* effect reduction in the levels of depression (Hedge g: −0.48) and anxiety (g: −0.47) with psychotherapy.

The utilization of psychotherapy has been suggested to impart beneficial effects on maternal quality of life as well. Eells^[93]^ reported that psychotherapeutic interventions could enhance the quality of life outcomes in addition to mental health. The authors reported that psychotherapy can effectively reduce the onset of behavior that exacerbates fatigue, pain, and insomnia. This, then, could allow enhancements in physical functioning and eventually the quality of life. Loughnan et al^[53]^ too reported that training with internet based cognitive behavioral training resulted in a better quality of life score for patients especially in terms of attentional concentration, personal esteem, and self-image. Our findings concerning the maternal quality of life outcomes are in line with the existing literature. In this present meta-analysis, we encountered a *small* beneficial effect of psychotherapy for enhancing the maternal quality of life (g: 0.19).

Finally, an important additional reason due to which the efficacy of psychotherapeutic interventions could have been enhanced, is that this intervention was delivered by paraprofessional nursing services. Grote et al^[70]^ for instance suggested that several cultural and environmental barriers can hinder the delivery of mental health services. The authors demonstrated that enhancing the cultural competence of the delivered intervention extended the benefits of a brief interpersonal psychotherapy intervention. Although not evaluated for the antenatal population group, we presume that the use of non-specialist staff such as nurses for delivering psychotherapeutic interventions could be an efficient way to enhance mental health prospects in an obstetric care unit. A previous randomized controlled trial by Espie et al^[94]^ had demonstrated the viability and efficacy of this approach. In this manner, not only the efficacy of the psychotherapy would be enhanced but also its effectiveness would be enhanced in developing countries where shortfalls in specialist personnel prevails the onset of antenatal depression and anxiety.^[95,96]^ Owing to the lack of pertinent literature it is difficult to interpret the comparative efficacy between culturally relevant care as compared to nurse administered therapies. Nevertheless, we presume that using paraprofessional nursing staff which has a similar cultural background as the patient would have an edge over nursing staff which does not share any cultural bond. Here, a similar cultural bondage between the caregiver and the expectant mother might help in alleviating pregnancy related anxiety while simultaneously removing communicational gaps.^[97]^ We strongly recommend future research to elucidate the comparative effectiveness of psychotherapeutic interventions being delivered by culturally relevant and irrelevant nursing staff for managing antenatal psychosomatic disorders.

Few limitations were present in this review. First, registration of this systematic review was not performed in a prospective registry such as PROSPERO. This might raise questions concerning the validity of this review.^[98]^ Second, we did not perform a literature search on renowned academic databases such as PsychInfo, and Sciencedirect. This too might raise questions concerning the outcomes of the review regarding the number of articles obtained after the review. Therefore, we would recommend future researchers to address this aspect in the future while performing a systematic search on a wide range of academic databases. Thirdly, a scarcity of statistical data in the included studies could have biased our interpretations concerning the influence of psychotherapy on maternal quality of life outcome. Here, the evaluation of quality of life outcome was performed only in two studies including a total of 65, 64 participants in the experimental and control groups, respectively. In this instance, the outcome due to a small sample size could possibly influence the results due to a type II error.^[99]^ We recommend future studies to address this paucity of data by evaluating the quality of life outcomes and sharing descriptive statistics in open access data repositories. Thirdly, because we incorporated a broad inclusion criterion in our review study, we were able to include a wide range of studies assessing different psychotherapeutic interventions with different assessment tools. Due to this, moderate heterogeneity, that is, 37%, 34% was observed in two of the meta-analysis reports analyzing the effects of psychotherapy on depression and anxiety, respectively. Therefore, we would strongly recommend our readers to carefully interpret these results.

In conclusion, this systematic review and meta-analysis provide a 1b level of evidence to support the use of psychotherapy to reduce depression, anxiety and enhance the maternal quality of life during the antenatal stage of pregnancy. The findings from the current meta-analyses can have widespread implications for developing best practice antenatal care approaches worldwide. In terms of practical applications, we strongly recommend antenatal care centers to employ the regular use of psychotherapeutic evaluations and therapies to alleviate depression, anxiety and enhance the maternal quality of life during the antenatal stage of pregnancy. Based on the existential resources antenatal care centers are recommended to assign a culturally relevant paraprofessional nursing staff to provide a one-on-on psychotherapeutic evaluation and therapy to alleviate any psychosomatic manifestation displayed by the mother. Moreover, in low- and middle-income countries where lack of resources adhere the capacity of medical staff to implement a one-on-one psychotherapy, the use of internet or mobile-application based psychotherapeutic approaches should be favored to reduce the burden of antenatal depression and anxiety.

## Author contributions

**Conceptualization:** Caixia Li, Dongyun Duan.

**Data curation:** Caixia Li, Xiaohua Sun, Qing Li, Qian Sun, Beibei Wu, Dongyun Duan.

**Formal analysis:** Xiaohua Sun, Qing Li, Qian Sun, Beibei Wu.

**Methodology:** Caixia Li, Xiaohua Sun, Qing Li, Qian Sun, Beibei Wu, Dongyun Duan.

**Project administration:** Qian Sun.

**Resources:** Xiaohua Sun, Qing Li, Beibei Wu, Dongyun Duan.

**Software:** Beibei Wu, Dongyun Duan.

**Supervision:** Dongyun Duan.

**Validation:** Caixia Li, Xiaohua Sun, Qing Li, Qian Sun, Beibei Wu, Dongyun Duan.

**Visualization:** Caixia Li, Xiaohua Sun, Qing Li, Qian Sun, Beibei Wu, Dongyun Duan.

**Writing – original draft:** Caixia Li.

**Writing – review & editing:** Dongyun Duan.
